# Artificial intelligence in prediabetes care: applications in screening, risk prediction, and lifestyle intervention

**DOI:** 10.3389/fendo.2026.1885905

**Published:** 2026-08-14

**Authors:** Runxuan Chen, Min Song, Tong Xiang

**Affiliations:** 1 School of Basic Medical Sciences, Cheeloo College of Medicine, Shandong University, Jinan, China; 2 Department of Neurology, Qilu Hospital of Shandong University (Qingdao), Qingdao, China; 3 Department of General Medicine, Qilu Hospital of Shandong University (Qingdao), Qingdao, China

**Keywords:** artificial intelligence, diabetes prevention, lifestyle intervention, machine learning, prediabetes, risk stratification, type 2 diabetes

## Abstract

Prediabetes is a highly prevalent intermediate metabolic state and a major public health target for preventing type 2 diabetes mellitus (T2DM). However, current approaches to prediabetes screening, risk stratification, and lifestyle management remain limited by inconsistent diagnostic definitions, incomplete case detection, heterogeneous progression risk, and the resource-intensive nature of conventional face-to-face prevention programmes. Artificial intelligence (AI), including machine learning, deep learning, explainable AI, and algorithm-driven digital interventions, is increasingly being explored as a tool to address these gaps. This narrative review used a structured search of PubMed, Web of Science Core Collection, and Embase for studies published from January 2010 to April 2026, selecting articles that evaluated AI-assisted or algorithm-driven approaches for prediabetes screening, progression risk prediction, or lifestyle intervention and reported relevant model performance, validation, or intervention outcomes. Current evidence indicates that AI-based models can improve discrimination beyond traditional risk scores by integrating routine clinical data, longitudinal electronic health records, continuous glucose monitoring profiles, wearable-derived behavioural signals, and emerging molecular biomarkers. Some externally validated models have shown clinically relevant performance for identifying individuals at high risk of progression and for guiding more targeted preventive strategies. In parallel, fully or semi-automated digital programmes delivered through mobile applications, web platforms, connected scales, and sensor-based feedback systems have demonstrated potential to support lifestyle change, improve engagement, and reduce reliance on labour-intensive counselling. Nevertheless, translation into routine care remains constrained by heterogeneity in prediabetes definitions, limited external validation across diverse populations, uncertain long-term effectiveness, geographical imbalance in evidence, privacy concerns, and the need for stronger governance frameworks. Overall, AI should be viewed as an assistive technology that may support earlier detection, more precise risk stratification, and scalable lifestyle management in prediabetes. Further multi-centre, prospective, and implementation-focused studies are needed to establish clinical utility, equity, safety, and cost-effectiveness.

## 1 Introduction

According to the 11th edition of the International Diabetes Federation (IDF) Diabetes Atlas, approximately 589 million adults aged 20 to 79 years were living with diabetes worldwide in 2025, and this number is projected to reach 853 million by 2050. Meanwhile, prediabetes, the intermediate metabolic state preceding type 2 diabetes mellitus (T2DM), is also highly prevalent and continues to rise. In 2021, the global age-adjusted prevalence of impaired glucose tolerance (IGT) among adults was estimated at 9.1% (about 464 million individuals), while impaired fasting glucose (IFG) affected 5.8% (about 298 million). Both are expected to increase to 10.0% and 6.5% by 2045, respectively (1). This continuous upward trend in diabetes and prediabetes imposes heavy pressure on healthcare resources, increases medical expenditures, and highlights the urgent need for effective early-stage management (2).

Prediabetes represents an early stage of dysglycemia and is widely recognized as a high-risk condition for progression to T2DM (3). Previous studies have shown that individuals with prediabetes progress to diabetes at an annual rate of approximately 5%-10% (4). Furthermore, up to 70% of people with prediabetes may eventually develop diabetes during their lifetime (5). Prediabetes is also associated with elevated risks of cardiovascular disease (CVD) and all-cause mortality (6–8). A meta-analysis including more than 10 million individuals reported that prediabetes was associated with a relative risk of 1.13 for all-cause mortality and 1.15 for composite cardiovascular events compared with normoglycemia (9). Importantly, randomized lifestyle intervention trials have demonstrated that structured programmes focusing on diet, physical activity, and weight reduction can reduce the risk of progression to diabetes by 58% (10). Despite this evidence, large gaps remain in the detection and management of prediabetes. Many individuals remain undiagnosed, and even those with a confirmed diagnosis often fail to receive timely, professional counseling or lifestyle guidance (11). These challenges are exacerbated by limited disease awareness, unequal distribution of medical resources, and insufficient knowledge of prediabetes among primary healthcare providers (PCPs) (12, 13).

With the rapid advancement of technology, artificial intelligence (AI) has become an important enabler in modern medicine, assisting in diagnostics, risk stratification, disease management, and outcome prediction (14). In oncology, AI-based imaging and pathology systems have achieved notable success, improving accuracy and efficiency in tumor detection and classification (15). Similarly, in chronic disease management, including hypertension and diabetes, AI has demonstrated considerable potential (16, 17). AI-driven tools can help optimize healthcare resource allocation, reduce variability in clinical decision-making, and provide personalized diagnostic and interventional strategies (18). These advantages suggest that AI may help overcome key barriers in prediabetes screening, diagnosis, and management by offering scalable and data-driven solutions.

This review aims to summarise and evaluate recent advances in the application of AI to prediabetes, covering screening and diagnostic assessment, progression risk prediction, lifestyle and digital interventions. The review also discusses current limitations and barriers to clinical implementation and outlines potential directions for future research and translation into routine care.

## 2 Diagnostic definitions and rationale for AI in prediabetes

### 2.1 Diagnostic definitions

Prediabetes is classically defined as an intermediate glycaemic state between normoglycaemia and T2DM, characterised by IFG, IGT, or elevated glycated haemoglobin (HbA1c) levels that remain below the diagnostic threshold for diabetes. The World Health Organization (WHO) defines IFG as fasting plasma glucose (FPG) 6.1–6.9 mmol/L, whereas the American Diabetes Association (ADA) adopts a lower FPG cut-off of 5.6–6.9 mmol/L. Both WHO and ADA define IGT as a 2-hour plasma glucose concentration of 7.8–11.0 mmol/L during a standard oral glucose tolerance test (OGTT). In addition, the ADA incorporates HbA1c values of 5.7–6.4% as a criterion for prediabetes, while WHO has not formally endorsed HbA1c for this purpose. The use of different diagnostic criteria across studies can therefore introduce heterogeneity in the populations classified as having prediabetes and complicate the interpretation and comparison of results (19).

### 2.2 Limitations of current screening and management approaches

Current approaches to identifying and managing prediabetes rely largely on episodic measurements of FPG, 2 hour OGTT glucose, or HbA1c, often combined with simple risk scores in primary care or community settings (20). While these tools are practical and widely used, they provide only a static snapshot of glycaemic status and can miss individuals with intermittent or postprandial abnormalities. In addition, conventional risk scores typically incorporate a limited set of variables, such as age, body mass index, family history, and blood pressure, and may show reduced performance when applied to populations that differ from the derivation cohort in ethnicity, socioeconomic background, or lifestyle patterns (21, 22). As highlighted in the Introduction, a substantial proportion of individuals with prediabetes remain undiagnosed. Moreover, traditional lifestyle intervention programmes for prediabetes largely depend on high-intensity, face-to-face counselling and health education (23). These programmes require considerable investments in personnel and financial resources, and although large clinical trials have demonstrated sustained improvements in glycaemic outcomes and reduced diabetes incidence with such interventions, their accessibility and scalability remain major limitations, particularly in resource-constrained settings (24, 25).

### 2.3 Rationale for applying artificial intelligence in prediabetes

**Figure 1:**
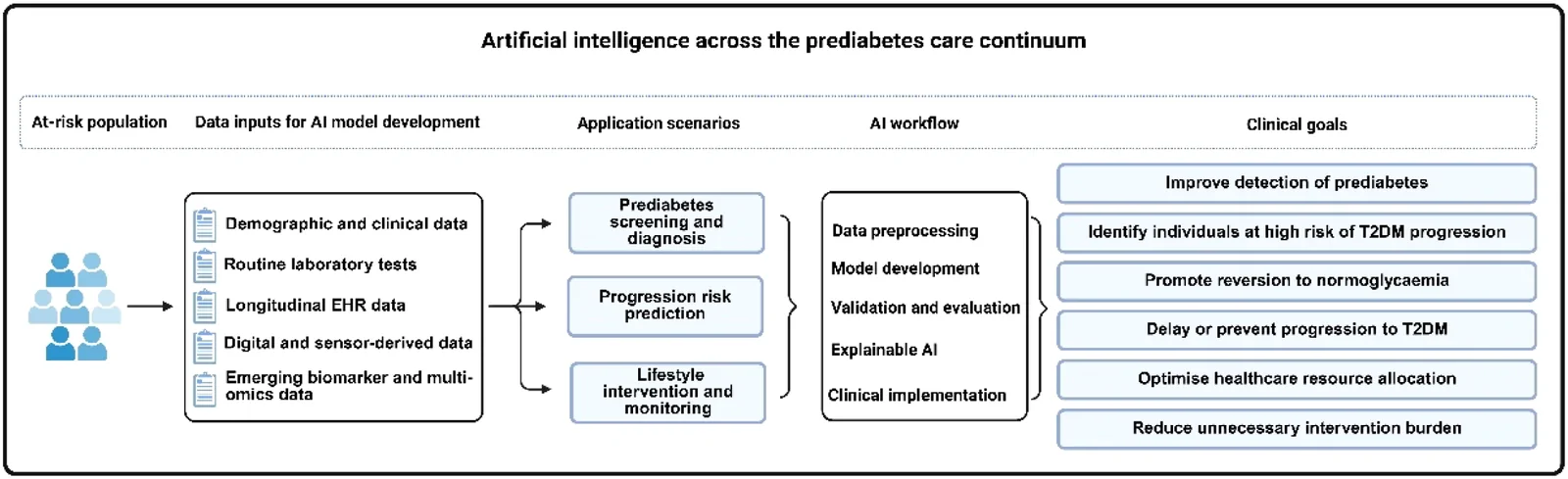
Artificial intelligence across the prediabetes care continuum. AI in prediabetes spans a complete workflow from at-risk populations and multimodal data inputs to data preprocessing, model development, validation and evaluation, explainable AI, and clinical implementation. These processes support three major application scenarios, namely prediabetes screening and diagnosis, progression risk prediction, and lifestyle intervention and monitoring, with the ultimate goals of improving detection, identifying individuals at high risk of progression to type 2 diabetes mellitus, promoting reversion to normoglycaemia, delaying disease progression, optimising healthcare resource allocation, and reducing unnecessary intervention burden.

AI, including machine learning (ML) and deep learning (DL) approaches, offers methodological tools that may help address several of the above challenges, as shown in **Figure 1**. Unlike traditional risk scores that rely on a small number of predefined predictors, AI models are capable of learning complex, non-linear relationships from high-dimensional data (26). In the context of prediabetes, this includes not only routine clinical and laboratory variables, but also longitudinal electronic health record (EHR) data, continuous glucose monitoring (CGM) profiles, physical activity and sleep metrics from wearable devices, and potentially imaging or multi-omics information (27–29). By integrating these heterogeneous data sources, AI can support more accurate risk prediction and finer-grained stratification within the broad category of prediabetes (30). In addition, AI-enabled decision-support systems provide an opportunity to embed such predictions into clinical workflows, guiding targeted screening, prioritising high-risk individuals for intensive lifestyle intervention, and monitoring response over time. Building on the same data streams, several fully automated digital programmes for prediabetes have been developed that integrate signals from activity sensors, CGM, and dietary intake analysis to tailor individualised goals and lifestyle recommendations (31, 32). These AI-driven interventions can adapt to users’ habits, enhance acceptability and engagement, and, as shown in recent trials, achieve glycaemic and behavioural outcomes that are non-inferior to those obtained with clinician- or coach-delivered lifestyle programmes (33). In addition, emerging evidence suggests that, compared with conventional intervention approaches, digital interventions may offer greater cost-effectiveness (34–36). Beyond these algorithm-driven digital interventions, generative AI (GAI) and large language models (LLMs) are emerging as a complementary direction for diabetes care. By generating, interpreting, and summarising natural language, these systems may support patient counselling, abnormal glucose triage, risk stratification, personalised lifestyle planning, clinical documentation, and primary care-based patient management (37, 38).

## 3 Methods

### 3.1 Review design

This article was designed as a narrative review. The aim was to synthesise current evidence on the application of AI in prediabetes care, with a focus on three major domains: screening and diagnostic assessment, risk stratification and prediction of progression to T2DM, and lifestyle or digital interventions.

### 3.2 Search strategy

A structured literature search was conducted in PubMed, Web of Science Core Collection, and Embase for articles published from January 2010 to April 2026. The final search was performed on 15 April 2026. Search terms combined concepts related to prediabetes and AI-based technologies. Reference lists of relevant reviews and key original studies were also manually screened to identify additional articles. The detailed database-specific search strings are provided in Supplementary material.

### 3.3 Study selection and evidence synthesis

Studies were considered relevant if they investigated AI-assisted, ML-based, DL-based, algorithm-driven, sensor-enabled, or digital health approaches for prediabetes screening, diagnostic assessment, risk stratification, prediction of progression to T2DM, or lifestyle and behavioural interventions. Studies involving individuals with prediabetes, IFG, IGT, intermediate hyperglycaemia, or populations at high risk of T2DM were considered for narrative synthesis. For prediction model studies, priority was given to those with large sample sizes, multicentre or population-based data sources, independent validation cohorts, clearly reported model performance, and clinically interpretable outcomes. Model development studies without external validation were generally not emphasised in the primary discussion unless they introduced novel biomarkers, emerging data modalities, innovative AI methods, or early-stage applications highly relevant to prediabetes. For digital intervention studies, randomised controlled trials, prospective studies, and pilot clinical trials were considered if they evaluated web-based platforms, smartphone applications, automated coaching systems, CGM, wearable devices, connected scales, or AI-enabled lifestyle interventions. Articles were not included in the main synthesis if they were unrelated to prediabetes or high-risk T2DM prevention, did not involve AI, ML, DL, algorithm-driven, digital, wearable, sensor-based, or biomarker-based approaches relevant to the review questions, lacked original data for the primary evidence synthesis, or did not report relevant model performance or intervention outcomes. Database searches identified a total of 1,508 records, including 440 from PubMed, 493 from Web of Science Core Collection, and 575 from Embase. After removal of 346 duplicates, 1162 records were screened by title and abstract. Of these, 1,026 records were excluded because they were not directly related to prediabetes or high-risk T2DM prevention, did not involve AI, ML, DL, algorithm-driven, digital, wearable, sensor-based, or biomarker-based approaches relevant to the review questions, were not original research articles, or did not report relevant model performance or intervention outcomes. Full texts of 136 articles were assessed for relevance, and 28 primary studies were ultimately included in the main narrative synthesis. The study selection process is summarised in **Figure 1** using an adapted PRISMA-style flow diagram. Review articles, systematic reviews, meta-analyses, guidelines, and consensus statements were additionally used to support background statements and contextualise the findings but were not counted as primary studies in the narrative synthesis.

## 4 AI-assisted diagnosis and risk stratification in prediabetes

As noted earlier, a substantial proportion of individuals with prediabetes remain undiagnosed, which delays early intervention and facilitates disease progression (39). Although traditional diagnostic and risk-stratification tools identify established risk factors, including sex, age, body mass index (BMI), waist circumference, race or ethnicity, and family history of diabetes, their predictive performance often remains modest (40, 41). AI, particularly ML and DL, offers distinctive advantages for constructing predictive models. By accommodating complex, high-dimensional data and capturing nonlinear relationships among variables, these approaches can uncover novel predictors and improve the discrimination of prediabetes beyond conventional linear models.

Several ML and DL tools for the diagnosis and risk stratification of prediabetes have been reported in recent years. We focus here on studies with large samples and independent external validation. The summary table is shown in **Table 1**. In a retrospective study of U.S. adults, Anderson and colleagues used a Bayesian analytics platform to develop a prediabetes prediction model (42). The final set of predictors included baseline glucose, insurance status, age, body temperature, alanine aminotransferase, BMI, C-reactive protein (CRP), triglycerides (TG), and high-density lipoprotein cholesterol (HDL-C). The model achieved areas under the receiver operating characteristic curve (AUCs) of 0.70 in the training cohort (n = 24331) and 0.72 in an external validation cohort (n = 189082). Notably, alanine aminotransferase, CRP, HDL-C, and body temperature emerged as previously underappreciated predictors, providing mechanistic hypotheses for future investigation (43–45).

**Table 1:** Summary of AI-assisted prediction models for prediabetes diagnosis and progression prediction.

Study	Prediction task	Dataset and population	Sample size	Algorithm/model	Input variables/data modality	Validation method	Main performance	Key limitations
Anderson et al.	Diagnosis / risk stratification	Retrospective EHR data from U.S. adults	Training: 24331; external validation: 189082	Bayesian analytics platform	Baseline glucose, insurance status, age, body temperature, ALT, BMI, CRP, TG, HDL-C	External validation	AUC 0.70 in training and 0.72 in external validation	Moderate discrimination; retrospective EHR design; possible coding and missing data bias
Choi et al.	Diagnosis / risk stratification	Korean population	Training and internal validation: 4685; external validation 4566	SVM and ANN	Age, sex, waist circumference, BMI, family history of diabetes, hypertension, alcohol intake	Internal and external validation	SVM AUC 0.761 and 0.731; ANN AUC 0.768 and 0.729; clinical tool AUC 0.734 and 0.712	Limited prespecified variables; prediabetes defined using FPG alone; no OGTT or HbA1c
Carrasco-Zanini et al.	Diagnosis / risk stratification	Population cohorts with OGTT and proteomic profiling	Internal validation: 2795; external validation: 1492	ML models using proteomic features	Aptamer-based plasma proteomics; 68-protein model; three-protein panel including RTN4R, CBPM, and GHR	Internal and external validation	68-protein model AUC 0.77; combined clinical and three-protein model AUC 0.80 internal and 0.69 external;	Proteomic assays may be costly and time-consuming; platform dependency; need standardisation and cost-effectiveness evaluation
Wang et al.	Diagnosis / risk stratification	Single-centre Chinese Han male cohort	Training: 781; internal validation: 336	RF and other ML models	Nine routinely available clinical variables	Internal validation	RF accuracy 96.7%, AUC 0.96, PPV 99.1%, NPV 95.6%	Single-centre study; restricted to middle-aged and older Han Chinese men; no broad external validation
Goodrich et al.	Diagnosis / risk stratification	Prospective multi-cohort study in overweight or obese Hispanic youth	143 participants; 37 progressed to prediabetes	ML-based feature selection and metabolite-augmented model	OGTT 2-hour post-load plasma metabolites and conventional risk factors	Training cohort and external validation cohort	Metabolite model AUC 0.80 vs 0.63 in training; 0.70 vs 0.62 in external validation	Small sample; population-specific; possible metabolite degradation during long-term storage
Li et al.	Diagnosis / risk stratification	China Health and Nutrition Survey	8277 participants	LASSO plus XGBoost; compared LR, RF, SVM, DT, NB, ANN	Age, TG, TC, BMI, ApoB, total protein, WBC, HDL-C, hypertension status	Internal test cohort	XGBoost AUC 0.978 in training and 0.939 in internal test set	Cross-sectional design; FPG-defined prediabetes; no prospective outcome prediction; broader external validation needed
Cahn et al.	Progression prediction	EHR data from the United Kingdom, Canada, and Israel	>850000 adults with prediabetes	Gradient boosted trees	69 variables from demographics, anthropometrics, laboratory values, comorbidities, and medication data	Internal validation and two external validation cohorts	1-year AUCs 0.865, 0.907, and 0.925; AUCs remained >0.80 for 3- and 5-year prediction	EHR missingness and coding variation; health-system-specific practice patterns; implementation feasibility not fully established
Zou et al.	Progression prediction	Chinese prediabetes cohort and BPRP population	Derivation/internal validation: 622; external validation: 478; application cohort: 1936	XGBoost-based ML-PR model	FPG, 2-hour OGTT glucose, HbA1c, HDL-C, TG	Internal and external validation; post-hoc intervention stratification	AUC 0.80 in internal validation and 0.80 in external validation; high-risk group derived greater benefit from intensive lifestyle plus pioglitazone	Moderate sample size for model development; population-specific; post-hoc intervention analysis
Jaeger et al.	Progression prediction	DPP and MESA cohorts	DPP: 2640; MESA: 2104	Cox proportional hazards model with treatment interactions	HbA1c, TG, FPG, BMI, age, sex, intervention arm	Internal validation in DPP; external validation in MESA	AUC 0.71 in DPP and 0.86 in MESA; model-guided allocation reduced number needed to treat	No combined lifestyle plus metformin arm; external AUC may reflect case-mix differences
Zhang et al.	Progression prediction	Two Chinese retrospective medical-centre cohorts	Primary cohort: 6578; external validation cohort: 2333	CatBoost	14 routinely available predictors selected by recursive feature elimination	Internal test cohort and external validation cohort	AUC 0.819 internal and 0.807 external	Retrospective design; external validation limited to Chinese medical-centre data; prospective utility not established

AI, artificial intelligence; ANN, artificial neural network; ALT, alanine aminotransferase; ApoB, apolipoprotein B; AUC, area under the receiver operating characteristic curve; BMI, body mass index; BPRP, Beijing Prediabetes Reversion Program; CGM, continuous glucose monitoring; CRP, C-reactive protein; DPP, Diabetes Prevention Program; DT, decision tree; EHR, electronic health record; FPG, fasting plasma glucose; HDL-C, high-density lipoprotein cholesterol; HbA1c, glycated haemoglobin; iIGT, isolated impaired glucose tolerance; LASSO, least absolute shrinkage and selection operator; LR, logistic regression; MESA, Multi-Ethnic Study of Atherosclerosis; ML, machine learning; NB, naive Bayes; NPV, negative predictive value; OGTT, oral glucose tolerance test; PPV, positive predictive value; RF, random forest; SHAP, SHapley Additive exPlanations; SVM, support vector machine; T2DM, type 2 diabetes mellitus; TC, total cholesterol; TG, triglycerides; WBC, white blood cell count; XGBoost, extreme gradient boosting.

In a Korean population, Choi and colleagues developed prediabetes prediction models using artificial neural networks (ANNs) and support vector machine (SVM) based on seven prespecified variables (age, sex, waist circumference, BMI, family history of diabetes, hypertension, and alcohol intake) (46). Compared with an existing clinical tool derived in Korean cohorts, the SVM and ANN models demonstrated superior performance in internal and external validation. Reported AUCs for SVM were 0.761 and 0.731, for ANNs were 0.768 and 0.729, and for the clinical tool were 0.734 and 0.712. The reliance on a limited, preselected feature set may have constrained the full potential of ML methods. In addition, the study defined prediabetes and diabetes using FPG alone without incorporating OGTT or HbA1c, which warrants cautious interpretation.

Because OGTT is time-consuming and less convenient in routine practice, many screening strategies for prediabetes and diabetes rely on FPG and HbA1c. This approach can miss individuals with isolated impaired glucose tolerance (iIGT). Evidence indicates that people with iIGT may have higher risks of complications and mortality than those with IFG, underscoring the need for improved identification strategies (47, 48). To address this gap, Carrasco-Zanini and colleagues applied an aptamer-based affinity assay for deep plasma proteomic profiling and combined the resulting features with ML models (49). The model built from 68 plasma proteins markedly outperformed a T2DM genetic risk score (AUC 0.77 vs 0.54) and exceeded the Cambridge T2DM Score (AUC 0.77 vs 0.75). After parsimony optimization, a three-protein model (RTN4R, CBPM, and GHR) achieved performance comparable to a clinical standard model that combined the Cambridge T2DM score with FPG and HbA1c. When the three-protein panel was integrated with the clinical standard model, the combined approach yielded the highest AUCs in both internal (n = 2795) and external (n = 1492) validation cohorts (0.80 and 0.69, respectively) and identified more than 30% additional cases of iIGT. These findings suggest that combining the three-protein panel with routine clinical information may help identify individuals who could benefit from further OGTT assessment for otherwise unrecognised iIGT. Nevertheless, the clinical translation of proteomic-based screening remains uncertain. Although the three-protein panel is more parsimonious than the original 68-protein model, its development relied on high-throughput aptamer-based proteomic profiling, which may be costly, time-consuming, and dependent on specialised laboratory platforms. Compared with FPG and HbA1c, such assays may be less scalable in primary care or population-level screening unless targeted protein measurements can be standardised, automated, and delivered at acceptable cost and turnaround time. Therefore, the clinical utility of this approach will depend not only on predictive performance, but also on analytical reproducibility, validated thresholds, workflow integration, and cost-effectiveness. More recently, Wang and colleagues developed iIGT prediction models using several ML algorithms (50). RF model based on nine routinely available clinical variables showed the most balanced performance in the internal validation cohort, achieving an accuracy of 96.7%, an AUC of 0.96, a PPV of 99.1%, and an NPV of 95.6%. However, the study was conducted at a single centre and included only middle-aged and older Han Chinese men. These features may have contributed to optimistic performance estimates and limit the generalisability of the findings.

Goodrich and colleagues recently conducted a prospective multi-cohort study in overweight or obese Hispanic youth to evaluate metabolic biomarkers as emerging predictors of prediabetes (51). Individuals with established prediabetes or diabetes were excluded. Baseline OGTT-2h plasma metabolites were quantified, and during follow-up 26.6% (37/143) progressed to prediabetes. Using ML-based feature selection, a metabolite-augmented model demonstrated superior performance compared with a model based on conventional risk factors. Reported AUCs were 0.80 vs. 0.63 in the training cohort and 0.70 vs. 0.62 in an external validation cohort. The study highlighted allylphenol sulfate and caprylic acid as candidate predictors with potential clinical relevance. The modest decline in external validation performance may reflect population heterogeneity and limited sample size. In addition, degradation of certain metabolites, such as fatty acids, during long-term storage could attenuate observed associations.

A barrier to clinical uptake of ML models is their limited transparency, which can erode clinician trust and impede implementation. Model-agnostic explainability tools, such as SHapley Additive exPlanations (SHAP), provide both global and local level attributions of feature contributions and allow users to visualize how specific values of predictors increase or decrease an individual’s estimated risk (52). In prediabetes applications, SHAP analyses help clinicians understand which variables drive predictions, where actionable thresholds may lie, and why two patients with similar risk profiles receive different outputs, thereby supporting clinical decision-making. Li and colleagues exemplified an interpretable model using China Health and Nutrition Survey data (53). They first applied least absolute shrinkage and selection operator (LASSO) regression to select nine readily obtainable predictors, namely age, TG, total cholesterol (TC), BMI, apolipoprotein B (ApoB), total protein (TP), white blood cell count (WBC), HDL-C, and hypertension status. They then compared several algorithms, among which extreme gradient boosting (XGBoost) achieved the best discrimination in the training (AUC 0.978) and internal test set (AUC 0.939) and outperformed logistic regression (LR), random forest (RF), SVM, decision tree (DT), Naive Bayes (NB), and ANNs. To enhance interpretability, they used SHAP to quantify and rank feature importance, identifying age, TG, and TC as leading contributors, and to derive clinically intuitive cut points from SHAP dependence plots, such as age > 53 years, BMI > 25 kg/m^2^, TC > 5.6 mmol/L, TG > 1.4 mmol/L, HDL-C < 1.2 mmol/L, and WBC > 6.2×10^9^/L. These analyses translate complex model behaviour into interpretable risk signals that may support targeted screening and counselling. However, the high discrimination reported in this study should be interpreted cautiously. The analysis was based on cross-sectional survey data and therefore cannot determine whether the model predicts future progression to prediabetes or T2DM. In addition, prediabetes was defined using FPG criteria, which may miss individuals with iIGT or HbA1c-defined prediabetes. Although a validation dataset was used, broader external validation across diverse populations and healthcare settings remains necessary.

## 5 AI-assisted prediction of progression from prediabetes to T2DM

The growing number of people living with diabetes places a substantial and escalating burden on health systems worldwide. In the United States, the total economic cost of diagnosed diabetes in 2022 was estimated at 412.9 billion USD, comprising 306.6 billion USD in direct medical expenditures and 106.3 billion USD in indirect costs related to reduced productivity, disability, and premature mortality (54). In China, which has the largest diabetes population, the total economic burden of diabetes in 2019 was estimated at approximately 156 billion USD, equivalent to about 1% of the national gross domestic product (55). Against this backdrop, preventing or delaying the transition from prediabetes to T2DM is a critical public health priority.

Clinical trials have consistently shown that early intervention in people with prediabetes can substantially reduce the risk of progression to T2DM and, in many cases, promote reversion to normoglycaemia (19). In the Diabetes Prevention Program (DPP), intensive lifestyle intervention focused on weight loss, dietary change, and increased physical activity reduced the incidence of diabetes by 58% compared with placebo, while metformin treatment achieved a 31% risk reduction in individuals with impaired glucose regulation (10). The Finnish Diabetes Prevention Study (DPS) likewise demonstrated that, during long-term follow-up, intensive lifestyle intervention produced more pronounced and sustained improvements in diet, physical activity, and cardiometabolic biomarkers, and significantly reduced the risk of developing diabetes (56). A recent systematic review concluded that lifestyle modification remains the most robust strategy to reverse prediabetes, although several pharmacological approaches can also induce remission in selected patients (57). Mechanistic and translational studies provide a biological basis for these observations. Early intensive lifestyle or dietary interventions can reduce ectopic fat in the liver and pancreas, improve insulin sensitivity, and partially restore β-cell function, thereby facilitating remission of dysglycaemia and altering the trajectory toward overt T2DM (58). Emerging evidence suggests that recovery of β-cell function plays a central role in remission of both prediabetes and early T2DM, whereas sustained hyperglycaemia and metabolic stress drive progressive β-cell failure and loss of glycaemic control (59–61). However, delivering intensive lifestyle or pharmacological interventions to all individuals with prediabetes is resource-intensive and may not be cost-effective, particularly in settings with constrained healthcare capacity. Large-scale programmes require substantial investments in trained personnel, infrastructure, and long-term follow-up, and may expose low-risk individuals to unnecessary treatment burden and societal costs (62). There is therefore a pressing need for robust tools and strategies to identify those people with prediabetes who are at highest risk of progressing to T2DM and would derive the greatest absolute benefit from intensive intervention, while allowing lower risk individuals to be managed with less intensive approaches (63). In the following sections, we review advances in AI-based risk prediction models that aim to refine progression risk stratification in prediabetes and to support more targeted and efficient prevention strategies.

One of the most influential examples is the ML model developed by Cahn and colleagues using EHRs from more than 850000 adults with prediabetes in the United Kingdom, Canada, and Israel (64). They employed a gradient boosted trees algorithm trained on The Health Improvement Network primary care database and then validated it in two independent cohorts from Canadian and Israeli health systems. Starting from hundreds of candidate features, the final model was implemented with 69 variables derived from 11 routinely collected signals, including demographics, anthropometrics, laboratory values, comorbidities, and medication data. For prediction of progression from prediabetes to T2DM within 1 year, the ML model consistently outperformed LR in the internal validation cohort and in both external validation cohorts, with AUCs of 0.865 vs. 0.778, 0.907 vs. 0.880, and 0.925 vs. 0.876, respectively. The model also maintained good discrimination for longer term outcomes, with AUCs remaining above 0.80 for prediction of incident diabetes over 3 and 5 years, without substantial attenuation. Other studies using static EMR snapshots have reported external AUCs as low as approximately 0.58, underscoring the limitations of approaches that do not exploit temporal information (42). By leveraging nearly a decade of longitudinal EHR data, Cahn and colleagues were able to capture trajectories, cumulative exposure, and repeated measurements of key risk factors, which likely contributed to the superior performance of their model and illustrated the added value of longitudinal data for risk stratification among individuals with prediabetes.

In a prospective study from China, Zou and colleagues developed an ML-based model to predict 1-year progression from prediabetes to diabetes and to guide interventions (65). The model was derived and internally validated in 622 adults with prediabetes, split 2:1 into a derivation and validation cohort. Several ML algorithms were compared, and an extreme gradient boosting (XGBoost) model was selected. To enhance usability, the final XGBoost model (ML-PR) included only five predictors: FPG, 2-hour post-load glucose during OGTT, HbA1c, HDL-C, and TG. In the internal validation cohort, ML-PR achieved an AUC of 0.80, outperforming the Framingham risk score (AUC 0.59) and the Cambridge Diabetes Risk Score (AUC 0.60). External validation in 478 participants from the conventional lifestyle plus placebo arm of the Beijing Prediabetes Reversion Program (BPRP) showed similar performance, with an AUC of 0.80. To assess risk stratification and clinical utility, ML-PR was applied to the entire BPRP population (n = 1,936), and participants were divided into low-, medium-, and high-risk groups according to tertiles of predicted progression probability. Progression and reversion rates over 1 and 3 years differed markedly across strata, confirming that the model identified groups with distinct long-term risk. Within each stratum, the four randomized interventions in BPRP were compared. In the low- and medium-risk groups, intensified lifestyle intervention, pioglitazone, or their combination did not provide clear additional benefit beyond conventional lifestyle counselling. In the high-risk group, however, the combination of intensified lifestyle intervention and pioglitazone reduced diabetes progression by about 50% and increased the probability of reversion to normoglycaemia by roughly one-third compared with conventional lifestyle alone. These findings suggest that an ML-PR based strategy can accurately identify roughly one third of individuals with prediabetes who are at particularly high risk of progression and who derive substantial benefit from combined lifestyle and pharmacological therapy, while allowing the remaining two thirds to avoid more intensive and potentially burdensome interventions (66). Such targeted risk stratification may improve the cost effectiveness of prevention programmes and reduce unnecessary exposure to medication related adverse effects (67). A recent study further tested the predictive performance of ML-PR in a U.S. cohort and achieved an AUC of 0.74 for predicting progression of prediabetes within 3 years; Risk stratification confirmed the increased risk of complications among high-risk patients and showed that the high-risk subgroups benefited more from lifestyle intervention (68).

In addition to ML-PR, a recent study by Jaeger and colleagues proposed a complementary approach that explicitly incorporates the effects of different preventive interventions on 3-year diabetes risk (69). Using data from the DPP, they fitted a Cox proportional hazards model in 2640 participants with prediabetes who had been randomised to intensive lifestyle intervention, metformin, or placebo. The model included six baseline predictors, namely HbA1c, TG, FPG, BMI, age, and sex, together with the intervention arm. Interaction terms between intervention and age, FPG, and BMI were added to estimate individualised intervention effects. In internal validation within the DPP, the model achieved an AUC of 0.71 for predicting 3-year progression to T2DM. External validation in 2104 adults with prediabetes from the Multi-Ethnic Study of Atherosclerosis (MESA) yielded an AUC of 0.86, suggesting very good discrimination in this independent cohort. Building on these individualised risk estimates, the authors showed that lifestyle intervention was the optimal option for 86% of DPP participants and 97% of MESA participants, whereas metformin was preferred in the remaining minority. Treatment allocation guided by the model reduced the number needed to treat to prevent one case of diabetes compared with uniform treat-all lifestyle or treat-all metformin strategies, highlighting the potential of such tools to support precision prevention. A key limitation is that the DPP did not include a combined intensive lifestyle plus metformin arm, so the model cannot estimate risk under a joint strategy, which remains an important clinical question. The higher AUC observed in MESA likely reflects differences in case mix, including an older and more heterogeneous validation cohort with lower baseline glycaemic levels.

Zhang and colleagues developed a 5-year progression model for Chinese adults with prediabetes using two retrospective cohorts from different medical centres (primary cohort n = 6578, external validation cohort n = 2333) (70). Using 14 routinely available predictors selected by recursive feature elimination, a CatBoost model achieved AUCs of 0.819 and 0.807 in the internal test cohort and external validation cohort, respectively, outperforming several alternative ML algorithms. SHAP highlighted FPG, HDL-C, ALT/AST ratio, BMI, age, and monocyte count as the most influential features, offering clinically intuitive targets for risk factor modification.

## 6 Digital, algorithm-driven, and AI-assisted lifestyle and behavioural interventions in prediabetes

Digital lifestyle interventions for prediabetes span a continuum from static educational tools and rule-based automation to dynamic AI-enabled systems. Static digital health tools typically deliver predefined educational content, reminders, or self-monitoring functions through web platforms, text messages, or mobile applications. Rule-based automated systems can tailor goals or feedback according to prespecified decision rules, such as user-reported diet, physical activity, weight change, or sensor-derived glucose patterns. In contrast, dynamic AI-enabled systems use ML, reinforcement learning, or adaptive algorithms to personalise the timing, frequency, content, or intensity of intervention components on the basis of baseline risk, behavioural patterns, engagement, and longitudinal data. This distinction is important because evidence supporting digital delivery or rule-based automation should not be interpreted as direct evidence for the effectiveness of AI.

Traditional diabetes prevention programmes rely heavily on face-to-face counselling and group education, which can limit scalability and reach (71). Digital and automated interventions may extend prevention support through smartphones, web platforms, connected scales, CGM, and wearable devices, while reducing clinician workload and standardising some aspects of behavioural support (40). Within this framework, AI has a more specific role: it may enable dynamic personalisation, identify early behavioural deterioration, integrate multimodal data streams, and trigger timely feedback or escalation. The following studies are therefore discussed according to their degree of adaptivity, ranging from static or rule-based digital programmes to more dynamic AI-enabled interventions.

Early digitally delivered interventions provide the foundation for later AI-assisted approaches but should be distinguished from AI systems. Web-based programmes, smartphone applications, email-based interventions, and text messaging platforms have been shown to support lifestyle change and improve metabolic outcomes in individuals with prediabetes (72, 73). Fully automated or semi-automated programmes delivered through email, web, and mobile interfaces have also been associated with modest but significant improvements in HbA1c, FPG, body weight, and diabetes risk scores compared with usual care in randomised or pilot studies (74–76). These interventions demonstrate the feasibility and scalability of remote lifestyle support, but many relied on predefined content, scheduled messaging, or rule-based tailoring rather than ML-based adaptation. Therefore, they are best interpreted as evidence for digital delivery and behavioural automation rather than direct evidence for AI efficacy. The summary table is shown in **Table 2**.

**Table 2:** Summary of digital, algorithm-driven, and AI-assisted lifestyle intervention studies in prediabetes.

Study	Design and population	Intervention type	AI or algorithmic component	Follow-up	Main outcomes	Key limitations
Block et al. / Alive-PD	Randomised controlled trial in adults with laboratory-confirmed prediabetes in a large U.S. health system	Fully automated, algorithm-driven behavioural intervention delivered by email, web portal, and mobile phone	Rule-based computer algorithms for personalised weekly goals, feedback, and problem-solving tips	6 months	Greater reductions in HbA1c, FPG, body weight, BMI, waist circumference, TG/HDL-C ratio, and predicted diabetes risk	Primarily rule-based rather than ML-based adaptation; comparator was usual care; long-term durability uncertain
Kitazawa et al.	Randomised controlled trial in adults at high risk of T2DM	Smartphone app plus intermittently scanned CGM	Sensor-enabled and rule-based feedback using CGM traces, diet, and physical activity information	12 weeks	Improved CGM-derived glycaemic metrics, modest weight loss, and better self-reported lifestyle behaviours	Feedback algorithms were mainly rule-based; short follow-up
Everett et al. / Sweetch	Pilot clinical trial in adults with prediabetes	Fully automated mobile health coaching platform	Proprietary ML algorithm tailoring frequency, timing, and content of push notifications according to baseline risk, preferences, engagement, and activity patterns	3 months	Increased physical activity, clinically meaningful weight loss, modest HbA1c reduction, and engagement-related outcome improvement	Pilot design; no randomised control group; short follow-up; limited causal inference
Cakmak et al. / TH-001	90-day randomised controlled trial in adults with prediabetes	Digital therapeutic delivered through mobile application	Structured lifestyle content, personalised prompts, and self-monitoring tools; degree of adaptive ML personalisation requires clarification	90 days	Greater reductions in HbA1c and body weight; higher engagement associated with greater glycaemic improvement	Mechanism appears mainly digital behavioural support unless adaptive ML is demonstrated; short duration; long-term outcomes unknown
Mathioudakis et al.	Randomised clinical trial in adults with prediabetes and overweight or obesity	AI-powered DPP-based lifestyle intervention delivered through mobile app and connected digital scale	Adaptive algorithms derived from Sweetch platform to personalise goals, messages, and feedback without ongoing human coaching	12 months	Non-inferior to human coaching for composite outcome; similar endpoint achievement, 31.7% vs 31.9%; engagement at least as high as human coaching	Modest sample size; single health system; one-year follow-up; longer-term durability and broader generalisability uncertain

AI, artificial intelligence; BMI, body mass index; CGM, continuous glucose monitoring; DPP, Diabetes Prevention Program; FPG, fasting plasma glucose; HbA1c, glycated haemoglobin; HDL-C, high-density lipoprotein cholesterol; ML, machine learning; T2DM, type 2 diabetes mellitus; TG, triglycerides.

Alive-PD is an important example of a fully automated, algorithm-driven behavioural intervention (77). In this randomised controlled trial, adults with laboratory-confirmed prediabetes in a large U.S. health system were assigned to a behavioural programme delivered through tailored emails, a web portal, and mobile phone interfaces, or to usual care without structured digital coaching. The programme used computer algorithms to set personalised weekly goals for diet, physical activity, and weight loss, provide feedback, and offer problem-solving tips based on participants’ self-reported behaviours. Over 6 months, the intervention group achieved significantly greater reductions in HbA1c (mean change, − 0.26% vs. − 0.18%, P < 0.001) and FPG (mean change, − 7.36 mg/dL vs. − 2.19 mg/dL; P < 0.001), larger decreases in body weight, BMI, waist circumference, and TG/HDL-C ratios, and a greater improvement in predicted diabetes risk compared with controls (5.00% vs. 1.41%, P < 0.001). Adherence to the programme was acceptable, with most participants maintaining engagement for a substantial part of the intervention period, supporting the feasibility of a fully automated approach in routine practice.

A complementary line of work has focused on sensor-enabled lifestyle support. In a 12-week randomised controlled trial from Japan, Kitazawa and colleagues enrolled adults at high risk of T2DM and allocated them to a smartphone application plus intermittently scanned CGM or to conventional care (32). The intervention displayed CGM traces, summarised glucose patterns, and provided automated feedback on diet and physical activity. Participants in the app plus isCGM arm showed greater improvements in time in range of blood glucose at 70 to 140 mg/dL (+ 31.5 minutes/day vs. – 2.6 minutes/day, P = 0.03), improvements in time spent in blood glucose < 70 mg/dL (+ 23.5 minutes/day vs. –8.9 minutes/day, P = 0.02), modest weight loss, and better self-reported lifestyle behaviours compared with controls. Because the feedback algorithms were primarily rule-based, this study is better interpreted as evidence that sensor-generated data can support personalised digital coaching and provide an infrastructure for future AI-driven interventions. TH-001 represents a structured digital therapeutic for adults with prediabetes (78). In a 90-day randomised controlled trial, participants were randomised to receive either standard care alone or standard care plus TH-001, which delivered lifestyle modification content, personalised prompts, and self-monitoring tools through a mobile application. The intervention group experienced greater reductions in HbA1c and body weight than the control group, and greater app engagement was associated with larger improvements in glycaemic control. These findings support the potential clinical value of structured digital therapeutics in prediabetes.

Sweetch more closely represents a dynamic AI-enabled intervention for adults with prediabetes (79). The platform uses a proprietary ML algorithm to tailor the frequency, timing, and content of push notifications according to each user’s baseline risk profile, preferences, engagement, and real-time activity patterns. Over 3 months, participants increased physical activity (+ 2.8 MET-hours per week, P = 0.02) and achieved clinically meaningful weight loss (– 1.6 kg, P < 0.001) and modest reductions in HbA1c (– 0.1%, P = 0.04), with higher app engagement associated with better outcomes. Although the study lacked a concurrent control group, it provided early evidence that an automated AI-driven mobile coach can support behavioural change in prediabetes. Nevertheless, the absence of randomisation and the short follow-up period limit causal inference regarding clinical effectiveness.

Across these studies, control conditions typically consisted of usual care or minimal lifestyle advice, and few trials directly compared automated or AI-enabled interventions with intensive, clinician-delivered lifestyle programmes. The trial by Mathioudakis and colleagues provides more direct evidence for a dynamic AI-powered lifestyle intervention (33). Adults with prediabetes and overweight or obesity were randomised to either an AI-powered DPP-based intervention delivered through a mobile app and connected digital scale or to a remote human coach-led DPP. The AI arm used adaptive algorithms derived from the Sweetch platform to personalise goals, messages, and feedback without ongoing involvement of human coaches. At 12 months, the AI intervention was non-inferior to the human coach-led programme with respect to the primary composite outcome, defined as maintaining an HbA1c level below 6.5% throughout the study and meeting at least one of the following criteria: at least 5% weight loss; at least 4% weight loss together with at least 150 minutes of physical activity per week; or an absolute reduction in HbA1c of at least 0.2 percentage points. The proportions of participants meeting the composite endpoint were similar in the two groups (31.7% vs. 31.9%). Engagement with the AI programme was at least as high as with human coaching, and model-guided interventions reduced reliance on labour-intensive counselling. However, the modest sample size, one-year follow-up, and recruitment from a single health system limit generalisability and underscore the need for larger, longer-term, and more diverse trials (80).

Overall, the current evidence should be interpreted along a spectrum of technological complexity. Static digital health tools and rule-based automated programmes show that lifestyle support can be delivered remotely and at scale, but they do not establish the specific added value of AI. Sensor-enabled interventions demonstrate how continuous behavioural and glycaemic data can be incorporated into feedback loops, although many current systems still rely on predefined rules. In contrast, dynamic AI-enabled interventions seek to adapt intervention content, timing, and intensity using individual-level data. Future studies should therefore clarify the degree of algorithmic adaptivity, report whether ML or rule-based logic is used, compare AI-driven systems with both usual care and high-quality human coaching, and evaluate long-term glycaemic outcomes, engagement, equity, safety, and cost-effectiveness.

## 7 Discussion

Overall, recent work has explored a broad range of applications of AI in prediabetes, spanning screening and diagnostic assessment, prediction of progression to T2DM, and delivery of lifestyle interventions. In screening and risk prediction, AI-based models have often demonstrated performance gains over conventional approaches, which likely reflects their ability to learn complex, non-linear relationships from high-dimensional and longitudinal data. In lifestyle management, multiple semi-automated and fully automated digital programmes have been developed to tailor goals, timing, and feedback to users’ preferences and behavioural patterns. Mobile and web-based platforms can lower barriers to participation relative to traditional in-person counselling, and several studies have reported improved engagement. Importantly, recent evidence also suggests that AI-enabled programmes can achieve outcomes comparable to human coach–delivered interventions in defined settings. These findings support the potential of AI to enhance the scalability and standardisation of behavioural support, but they also require careful interpretation because algorithmic performance, clinical applicability, and real-world benefit are not equivalent.

Comparisons across these models should be made cautiously because the reviewed studies differed substantially in diagnostic definitions, target outcomes, input variables, and validation designs. Some models identified prevalent prediabetes defined mainly by FPG, whereas others focused on iIGT detected by OGTT or predicted future progression from prediabetes to T2DM. These distinctions are clinically important because FPG-defined prediabetes, HbA1c-defined prediabetes, IGT, and iIGT represent overlapping but not identical metabolic states, with different underlying pathophysiology and risk profiles (81). As a result, a high AUC in one setting does not necessarily indicate superior performance in another setting with a different case definition, disease prevalence, or outcome window. Input data also influence both discrimination and feasibility. Models based on simple clinical variables are easier to implement in primary care but may miss postprandial dysglycaemia or biological heterogeneity; models incorporating proteomic, metabolomic, CGM, wearable, or longitudinal EHR data may capture richer signals but require stronger data infrastructure, assay standardisation, and external validation (82). Therefore, algorithmic performance metrics should not be interpreted in isolation. Calibration, clinical utility, decision-curve analysis, cost-effectiveness, workflow integration, and evidence that model-guided decisions improve patient outcomes are all necessary before AI tools can be considered clinically beneficial.

The reviewed studies also indicate that the clinical value of an AI model depends not only on the algorithm used, but also on the clinical question, data structure, and implementation setting. Simpler approaches, such as LR or Cox regression, remain useful when the number of predictors is limited, the relationships between predictors and outcomes are relatively stable, and transparent risk estimation is prioritised. SVMs and ANNs may improve discrimination over conventional risk scores in some screening tasks, but their advantage can be constrained when they are trained on a small number of prespecified variables (83). Tree-based ensemble methods, including RF, gradient boosting, XGBoost, and CatBoost, appear particularly suitable for tabular clinical data because they can model non-linear associations, handle interactions among metabolic variables, and accommodate mixed predictor types (84). These features may explain their strong performance in several prediabetes screening and progression prediction studies. However, improved discrimination does not automatically translate into clinical benefit, especially when models lack calibration assessment, external validation, decision-curve analysis, or evidence that model-guided care changes management decisions or outcomes. Longitudinal EHR-based models provide a further advantage by capturing trajectories and cumulative exposure over time, but they require high-quality repeated measurements and may be vulnerable to missingness, coding variation, and health-system-specific practice patterns (85). Multi-omics and sensor-based models may capture biological or behavioural signals that are not reflected in routine clinical variables, but their applicability is limited by cost, platform dependency, data harmonisation requirements, and uncertain scalability (86, 87). Therefore, the most appropriate algorithm is unlikely to be universal; it should be selected according to the intended use case, available data, interpretability requirements, resource setting, and the level of clinical risk associated with the decision being supported.

In recent years, an increasing number of studies have used ML and DL algorithms to identify determinants of prediabetes and to develop models for its detection and risk stratification. These studies suggest that novel molecular markers and data generated by emerging sensing technologies may provide additional insights into the diagnosis and management of prediabetes, particularly when integrated with conventional clinical variables. Fathimal and colleagues developed a non-invasive optical sensing device designed to capture vascular optical signals in real time for glucose status screening (88). In a small validation cohort, ensemble bagged trees and RF classifiers achieved accuracies of 86.67%, 87.50%, and 85.71%, and 80.00%, 87.50%, and 71.43%, respectively, for classifying normoglycaemia, prediabetes, and diabetes. Although these findings support the feasibility of optical signal-based glucose status classification, the small sample size and early-stage validation limit conclusions regarding clinical applicability. Other studies have explored ECG-derived signals for non-invasive glycaemic assessment. Chiu and colleagues developed an SVM model using ECG features extracted from individual heartbeats, reporting an AUC of 0.92, with sensitivity and specificity of 0.92 and 0.84, respectively, for detecting abnormal glucose status (89). Similarly, IGRNet, a DL model based on 5-second 12-lead ECG recordings, was developed for non-invasive, real-time diagnosis of prediabetes (90). These findings indicate that ECG signals may contain latent information related to impaired glucose regulation, although their diagnostic value requires further validation across larger and more diverse populations. Beyond optical and ECG-based approaches, other non-invasive data modalities have also been investigated. Oreskovic and colleagues developed voice-based models for prediabetes prediction (91). Fazle Karim and colleagues tested a novel radiofrequency glucose sensor for noninvasive real-time glucose monitoring (92). Niu and colleagues analysed longitudinal glycomic data from the KORA F4 and FF4 cohorts and identified 19 key plasma N-glycan traits, including GP32, GP22, GP34, and GP18, as predictors of impaired glucose regulation (93). Several exploratory studies have reported additional emerging approaches. For example, Kaup and colleagues developed an AI model based on retinal vascular features and reported discrimination for diabetes and prediabetes (94). Singh and colleagues identified DNA methylation signatures in blood cells that may support risk stratification for diabetes progression and complications among individuals with prediabetes (95). Inflammatory markers (96), breath biomarkers (97, 98), tear proteomic profiles (99), urinary peptidome markers (100), and gut microbiome features have also been explored as potential biomarkers (101, 102). Together, these studies broaden the data landscape for prediabetes research and suggest that dysglycaemia may be reflected not only in conventional glycaemic indices but also in vascular, electrophysiological, acoustic, metabolic, epigenetic, proteomic, peptidomic, and microbial signals. However, most of these approaches remain at an early stage, and their clinical translation is constrained by small or selected study populations, limited external validation, platform dependency, uncertain reproducibility, and insufficient evidence regarding implementation cost and scalability. Despite these uncertainties, multimodal and multi-omics models based on emerging biomarkers may offer important advantages when they are integrated with routine clinical data (103, 104). By combining complementary biological, physiological, behavioural, and clinical information, such models may improve discrimination, capture heterogeneous pathways of dysglycaemia, and support more refined phenotyping of individuals with prediabetes (105). In particular, advances in non-invasive glucose monitoring and sensor-based assessment may reduce patient burden, improve the convenience and acceptability of glucose monitoring, enhance adherence to repeated measurements, and provide real-time feedback for evaluating lifestyle or pharmacological interventions. AI can serve as a bridge between conventional clinical indicators and emerging biomarker or sensor-derived data by integrating heterogeneous inputs into clinically interpretable risk estimates. Accurate and convenient glycaemic monitoring is therefore a foundational component of prediabetes care, because it supports progression risk assessment, complication prediction, intervention evaluation, and timely adjustment of preventive strategies.

GAI and LLMs represent an emerging but still underexplored direction in prediabetes care. Unlike conventional ML models that primarily perform classification or risk prediction, LLMs may support natural language interaction, patient education, lifestyle counselling, clinical note summarisation, and extraction of behavioural or social determinants from unstructured health records (106). These capabilities are particularly relevant to prediabetes, where long-term lifestyle modification and sustained patient engagement are central to prevention. However, direct evidence in prediabetes populations remains sparse. Most available evidence comes from general medical question-answering, diabetes education, and clinical decision-support studies rather than prospective trials in individuals with prediabetes (106, 107). Current LLMs may generate plausible but inaccurate or insufficiently individualised recommendations, and their performance can be affected by prompt design, input order, guideline updates, and patient complexity (108–110). Therefore, LLMs should currently be viewed as supervised assistive tools rather than autonomous systems for prediabetes screening or management. A promising future direction is the development of prediabetes-specific LLMs built through domain adaptation, fine-tuning, retrieval-augmented generation, or integration with validated clinical guidelines, nutrition databases, EHR context, and structured risk prediction models. Compared with general-purpose LLMs, such domain-adapted systems may provide more context-specific, guideline-concordant, and clinically actionable responses to questions related to glycaemic monitoring, lifestyle modification, and long-term self-management (111). Nevertheless, these potential advantages remain insufficiently validated in prediabetes care. Future studies should evaluate LLM-based interventions in well-defined prediabetes cohorts, assess guideline concordance, factual accuracy, safety, patient engagement, metabolic outcomes, equity, privacy, and cost-effectiveness, and compare them with established human coach-led or algorithm-driven digital prevention programmes (112).

Several limitations should be considered when interpreting the current literature. First, diagnostic definitions of prediabetes are not uniform across studies, including variation in glycaemic thresholds and the use of HbA1c for case ascertainment. This inconsistency introduces heterogeneity in study populations and complicates cross-study comparisons of model performance and intervention effects. Second, accumulating mechanistic evidence indicates that prediabetes is biologically heterogeneous, with distinct trajectories shaped by varying degrees of insulin resistance, beta-cell dysfunction, and tissue-specific dysregulation (113, 114). For example, in a recent large-scale study in a Chinese population, Lin and colleagues applied the Discriminative Dimensionality Reduction Tree (DDRTree) algorithm to identify four prediabetes phenotypes (115). Among these, phenotype 4, characterised by hyperglycaemia, obesity, elevated triglycerides, and increased liver enzyme levels, showed the highest risk of progression to T2DM, whereas phenotype 3 was associated with the highest risk of chronic kidney disease. These findings further support the view that a one-size-fits-all approach to risk stratification or intervention may dilute benefit for high-risk subgroups, underscoring the need for refined phenotyping and targeted strategies for those most likely to progress (116). Third, the geographical distribution of evidence is uneven. Most AI studies in prediabetes have been developed and validated in high-income settings, while data from low- and middle-income countries and other resource-limited regions remain comparatively scarce. Broader analyses of AI in diabetes care have similarly highlighted under-representation of diverse populations, limited external validation, and concerns regarding generalisability across health systems. In lower-resource settings, implementation may be further constrained by weaker digital infrastructure, variable data quality, and lower uptake of digital health tools (117). Together, these gaps raise important concerns about transportability, fairness, and real-world feasibility, and underscore the need for broader geographical representation and implementation-focused validation studies (118, 119). Responsible implementation of AI in prediabetes care will also require attention to explainability, privacy, bias, accountability, and regulatory oversight. Explainable AI is particularly important because screening and prevention decisions are preference-sensitive and often involve individuals who are asymptomatic. Clinicians and patients need to understand why a model classifies someone as high risk, which factors are modifiable, and how the prediction should guide subsequent testing or intervention. Methods such as SHAP can improve transparency by identifying influential predictors and patient-specific risk drivers, but explainability outputs should be interpreted as decision-support information rather than definitive causal explanations. Moreover, high model accuracy does not eliminate the need for clinical judgement, because poorly calibrated or insufficiently validated models may lead to overdiagnosis, unnecessary anxiety, inappropriate escalation of intervention, or missed opportunities for prevention. Data governance is another central challenge. AI models for prediabetes increasingly rely on multiple levels of information, all of which raise concerns about consent, secondary data use, unauthorised access, data leakage, and patient re-identification. Such concerns may reduce willingness to share data, thereby limiting collaborative research and slowing progress in model development and validation (120, 121). Clear governance structures are also needed to define responsibility for model updates, human oversight, documentation, auditability, and management of errors or unintended harms. In this context, regulatory evaluation should focus not only on technical performance but also on safety, fairness, transparency, workflow integration, and post-deployment surveillance (122).

Looking ahead, rapid advances in algorithms, along with improved access to longitudinal and multimodal data, may expand the role of AI in prediabetes research and prevention (123, 124). Multimodal AI models that integrate routine clinical variables, longitudinal EHRs, CGM profiles, wearable-derived behavioural signals, dietary information, imaging data, and molecular biomarkers may enable more precise identification of high-risk individuals and more refined phenotyping of heterogeneous prediabetes subgroups (125, 126). In parallel, foundation models trained on large-scale clinical, sensor-derived, imaging, or multi-omics datasets may provide reusable representations that can be adapted to multiple prediabetes tasks, including screening, progression prediction, intervention selection, and treatment response monitoring. GAI and LLMs may further support personalised prediabetes management by translating model outputs into patient-friendly explanations, generating tailored lifestyle education materials, summarising longitudinal records, and assisting primary care clinicians in follow-up planning and risk communication. However, these approaches will require rigorous evaluation of factual accuracy, calibration, clinical utility, bias, privacy protection, human oversight, and cost-effectiveness before they can be incorporated into routine care. Finally, stronger international collaboration is essential to support large, multi-centre, and diverse cohorts, which are required for robust model development, external validation, assessment of generalisability, and evaluation of implementation in real-world healthcare settings (127).

## 8 Conclusion

AI is reshaping prediabetes prevention and management by extending its role across screening, diagnostic assessment, progression risk prediction, and lifestyle intervention. Current evidence suggests that AI-based models may improve risk stratification beyond conventional tools by integrating routine clinical data, longitudinal EHRs, wearable-derived signals, CGM profiles, and emerging biomarker information, while explainable AI may enhance interpretability and support targeted screening and counselling. Progression prediction models may help identify individuals at highest risk of developing T2DM and guide more efficient allocation of intensive lifestyle or pharmacological interventions, whereas automated and AI-enabled digital programmes may provide scalable behavioural support when access to intensive face-to-face counselling is limited. Clinically, these tools should currently be used as clinician-supervised adjuncts rather than standalone systems, with priority given to models that use accessible data, have been externally validated in relevant populations, report calibration and clinical utility, and provide interpretable outputs for shared decision-making. Future research should prioritise standardised diagnostic definitions, diverse multi-centre validation, fairness and bias assessment, decision-curve and cost-effectiveness analyses, long-term intervention trials, and prospective implementation studies to determine whether AI-guided screening, risk stratification, and lifestyle support improve reversion to normoglycaemia, prevention of T2DM, patient engagement, safety, equity, and healthcare resource use. Further evaluation of multimodal AI, foundation models, and GAI or LLM-based tools should also be conducted under clinician supervision, with clear attention to privacy, governance, and post-deployment monitoring.
