# Supplementary material for: Artificial intelligence in prediabetes care: applications in screening, risk prediction, and lifestyle intervention
Source: Front Endocrinol (Lausanne). 2026 Aug 14;17:1885905. doi: 10.3389/fendo.2026.1885905 (PMC13522911; doi:10.3389/fendo.2026.1885905)
Supplement: Supplementary file 1 [file Table1.docx]

**Search strategy:**

**Web of Science：**

#1

TS=(

prediabet*

OR pre-diabet*

OR "impaired fasting glucose"

OR "impaired glucose tolerance"

OR "isolated impaired fasting glucose"

OR "isolated impaired glucose tolerance"

OR "glucose intolerance"

OR "intermediate hyperglyc*"

OR "intermediate dysglyc*"

)

#2

TS=(

"artificial intelligence"

OR "machine learning"

OR "deep learning"

OR "machine intelligence"

OR "neural network*"

OR "artificial neural network*"

OR "convolutional neural network*"

OR "recurrent neural network*"

OR "support vector machine*"

OR "random forest*"

OR "gradient boost*"

OR "extreme gradient boost*"

OR XGBoost

OR CatBoost

OR LightGBM

OR AdaBoost

OR "decision tree*"

OR "Bayesian network*"

OR "ensemble learning"

OR "k-nearest neighbor*"

OR "k nearest neighbor*"

OR "k-nearest neighbour*"

OR "k nearest neighbour*"

OR "naive Bayes"

OR "naïve Bayes"

OR "data mining"

OR "predictive analytics"

OR "computer-aided diagnosis"

OR "computer assisted diagnosis"

OR "clinical decision support"

OR "clinical decision support system*"

OR "explainable artificial intelligence"

OR "explainable AI"

OR "interpretable machine learning"

OR "natural language processing"

OR "large language model*"

OR "generative artificial intelligence"

OR "generative AI"

OR ChatGPT

OR "foundation model*"

OR "transformer model*"

OR "reinforcement learning"

OR "expert system*"

OR "knowledge-based system*"

OR "rule-based system*"

)

**Pubmed：**

(

"Prediabetic State"[Mesh]

OR "Glucose Intolerance"[Mesh]

OR prediabet*[tiab]

OR pre-diabet*[tiab]

OR "impaired fasting glucose"[tiab]

OR "impaired glucose tolerance"[tiab]

OR "isolated impaired fasting glucose"[tiab]

OR "isolated impaired glucose tolerance"[tiab]

OR "glucose intolerance"[tiab]

OR "intermediate hyperglyc*"[tiab]

OR "intermediate dysglyc*"[tiab]

)

AND

(

"Artificial Intelligence"[Mesh]

OR "Machine Learning"[Mesh]

OR "Clinical Decision Support Systems"[Mesh]

OR "Natural Language Processing"[Mesh]

OR "artificial intelligence"[tiab]

OR "machine learning"[tiab]

OR "deep learning"[tiab]

OR "machine intelligence"[tiab]

OR "neural network*"[tiab]

OR "artificial neural network*"[tiab]

OR "convolutional neural network*"[tiab]

OR "recurrent neural network*"[tiab]

OR "support vector machine*"[tiab]

OR "random forest*"[tiab]

OR "gradient boost*"[tiab]

OR "extreme gradient boost*"[tiab]

OR XGBoost[tiab]

OR CatBoost[tiab]

OR LightGBM[tiab]

OR AdaBoost[tiab]

OR "decision tree*"[tiab]

OR "Bayesian network*"[tiab]

OR "ensemble learning"[tiab]

OR "k-nearest neighbor*"[tiab]

OR "naive Bayes"[tiab]

OR "data mining"[tiab]

OR "predictive analytics"[tiab]

OR "computer-aided diagnosis"[tiab]

OR "computer assisted diagnosis"[tiab]

OR "clinical decision support"[tiab]

OR "explainable artificial intelligence"[tiab]

OR "explainable AI"[tiab]

OR "interpretable machine learning"[tiab]

OR "natural language processing"[tiab]

OR NLP[tiab]

OR "large language model*"[tiab]

OR "generative artificial intelligence"[tiab]

OR "generative AI"[tiab]

OR ChatGPT[tiab]

OR "foundation model*"[tiab]

OR "transformer model*"[tiab]

OR "reinforcement learning"[tiab]

)

**Embase：**

(

'prediabetes'/exp

OR 'impaired fasting glucose'/exp

OR 'impaired glucose tolerance'/exp

OR prediabet*:ti,ab,kw

OR pre-diabet*:ti,ab,kw

OR 'impaired fasting glucose':ti,ab,kw

OR 'impaired glucose tolerance':ti,ab,kw

OR 'isolated impaired fasting glucose':ti,ab,kw

OR 'isolated impaired glucose tolerance':ti,ab,kw

OR 'glucose intolerance':ti,ab,kw

OR 'intermediate hyperglyc*':ti,ab,kw

OR 'intermediate dysglyc*':ti,ab,kw

)

AND

(

'artificial intelligence'/exp

OR 'machine learning'/exp

OR 'deep learning'/exp

OR 'natural language processing'/exp

OR 'artificial intelligence':ti,ab,kw

OR 'machine learning':ti,ab,kw

OR 'deep learning':ti,ab,kw

OR 'machine intelligence':ti,ab,kw

OR 'neural network*':ti,ab,kw

OR 'artificial neural network*':ti,ab,kw

OR 'convolutional neural network*':ti,ab,kw

OR 'recurrent neural network*':ti,ab,kw

OR 'long short-term memory':ti,ab,kw

OR LSTM:ti,ab,kw

OR 'support vector machine*':ti,ab,kw

OR 'random forest*':ti,ab,kw

OR 'gradient boost*':ti,ab,kw

OR 'extreme gradient boost*':ti,ab,kw

OR XGBoost:ti,ab,kw

OR CatBoost:ti,ab,kw

OR LightGBM:ti,ab,kw

OR AdaBoost:ti,ab,kw

OR 'decision tree*':ti,ab,kw

OR 'Bayesian network*':ti,ab,kw

OR 'ensemble learning':ti,ab,kw

OR 'k-nearest neighbor*':ti,ab,kw

OR 'k nearest neighbor*':ti,ab,kw

OR 'k-nearest neighbour*':ti,ab,kw

OR 'k nearest neighbour*':ti,ab,kw

OR 'naive Bayes':ti,ab,kw

OR 'naïve Bayes':ti,ab,kw

OR 'data mining':ti,ab,kw

OR 'predictive analytics':ti,ab,kw

OR 'computer-aided diagnosis':ti,ab,kw

OR 'computer assisted diagnosis':ti,ab,kw

OR 'clinical decision support':ti,ab,kw

OR 'clinical decision support system*':ti,ab,kw

OR 'expert system*':ti,ab,kw

OR 'knowledge-based system*':ti,ab,kw

OR 'rule-based system*':ti,ab,kw

OR 'explainable artificial intelligence':ti,ab,kw

OR 'explainable AI':ti,ab,kw

OR 'interpretable machine learning':ti,ab,kw

OR 'natural language processing':ti,ab,kw

OR 'large language model*':ti,ab,kw

OR 'generative artificial intelligence':ti,ab,kw

OR 'generative AI':ti,ab,kw

OR ChatGPT:ti,ab,kw

OR 'generative pre-trained transformer*':ti,ab,kw

OR 'foundation model*':ti,ab,kw

OR 'transformer model*':ti,ab,kw

OR 'reinforcement learning':ti,ab,kw

)

**Supplementary Figure:**

Figure S1: PRISMA flow diagram.
