# Supplementary material for: Artificial intelligence in prediabetes care: applications in screening, risk prediction, and lifestyle intervention
Source: Front Endocrinol (Lausanne). 2026 Aug 14;17:1885905. doi: 10.3389/fendo.2026.1885905 (PMC13522911; doi:10.3389/fendo.2026.1885905)
Supplement: Supplementary file 2 [file Table2.docx]

**Tables:**

**Table 1. Summary of AI-assisted prediction models for prediabetes diagnosis and progression prediction**

| **Study** | **Prediction task** | **Dataset and population** | **Sample size** | **Algorithm/model** | **Input variables/data modality** | **Validation method** | **Main performance** | **Key limitations** |
| --- | --- | --- | --- | --- | --- | --- | --- | --- |
| Anderson et al. | Diagnosis / risk stratification | Retrospective EHR data from U.S. adults | Training: 24331; external validation: 189082 | Bayesian analytics platform | Baseline glucose, insurance status, age, body temperature, ALT, BMI, CRP, TG, HDL-C | External validation | AUC 0.70 in training and 0.72 in external validation | Moderate discrimination; retrospective EHR design; possible coding and missing data bias |
| Choi et al. | Diagnosis / risk stratification | Korean population | Training and internal validation: 4685; external validation 4566 | SVM and ANN | Age, sex, waist circumference, BMI, family history of diabetes, hypertension, alcohol intake | Internal and external validation | SVM AUC 0.761 and 0.731; ANN AUC 0.768 and 0.729; clinical tool AUC 0.734 and 0.712 | Limited prespecified variables; prediabetes defined using FPG alone; no OGTT or HbA1c |
| Carrasco-Zanini et al. | Diagnosis / risk stratification | Population cohorts with OGTT and proteomic profiling | Internal validation: 2795; external validation: 1492 | ML models using proteomic features | Aptamer-based plasma proteomics; 68-protein model; three-protein panel including RTN4R, CBPM, and GHR | Internal and external validation | 68-protein model AUC 0.77; combined clinical and three-protein model AUC 0.80 internal and 0.69 external; | Proteomic assays may be costly and time-consuming; platform dependency; need standardisation and cost-effectiveness evaluation |
| Wang et al. | Diagnosis / risk stratification | Single-centre Chinese Han male cohort | Training: 781; internal validation: 336 | RF and other ML models | Nine routinely available clinical variables | Internal validation | RF accuracy 96.7%, AUC 0.96, PPV 99.1%, NPV 95.6% | Single-centre study; restricted to middle-aged and older Han Chinese men; no broad external validation |
| Goodrich et al. | Diagnosis / risk stratification | Prospective multi-cohort study in overweight or obese Hispanic youth | 143 participants; 37 progressed to prediabetes | ML-based feature selection and metabolite-augmented model | OGTT 2-hour post-load plasma metabolites and conventional risk factors | Training cohort and external validation cohort | Metabolite model AUC 0.80 vs 0.63 in training; 0.70 vs 0.62 in external validation | Small sample; population-specific; possible metabolite degradation during long-term storage |
| Li et al. | Diagnosis / risk stratification | China Health and Nutrition Survey | 8277 participants | LASSO plus XGBoost; compared LR, RF, SVM, DT, NB, ANN | Age, TG, TC, BMI, ApoB, total protein, WBC, HDL-C, hypertension status | Internal test cohort | XGBoost AUC 0.978 in training and 0.939 in internal test set | Cross-sectional design; FPG-defined prediabetes; no prospective outcome prediction; broader external validation needed |
| Cahn et al. | Progression prediction | EHR data from the United Kingdom, Canada, and Israel | >850000 adults with prediabetes | Gradient boosted trees | 69 variables from demographics, anthropometrics, laboratory values, comorbidities, and medication data | Internal validation and two external validation cohorts | 1-year AUCs 0.865, 0.907, and 0.925; AUCs remained >0.80 for 3- and 5-year prediction | EHR missingness and coding variation; health-system-specific practice patterns; implementation feasibility not fully established |
| Zou et al. | Progression prediction | Chinese prediabetes cohort and BPRP population | Derivation/internal validation: 622; external validation: 478; application cohort: 1936 | XGBoost-based ML-PR model | FPG, 2-hour OGTT glucose, HbA1c, HDL-C, TG | Internal and external validation; post-hoc intervention stratification | AUC 0.80 in internal validation and 0.80 in external validation; high-risk group derived greater benefit from intensive lifestyle plus pioglitazone | Moderate sample size for model development; population-specific; post-hoc intervention analysis |
| Jaeger et al. | Progression prediction | DPP and MESA cohorts | DPP: 2640; MESA: 2104 | Cox proportional hazards model with treatment interactions | HbA1c, TG, FPG, BMI, age, sex, intervention arm | Internal validation in DPP; external validation in MESA | AUC 0.71 in DPP and 0.86 in MESA; model-guided allocation reduced number needed to treat | No combined lifestyle plus metformin arm; external AUC may reflect case-mix differences |
| Zhang et al. | Progression prediction | Two Chinese retrospective medical-centre cohorts | Primary cohort: 6578; external validation cohort: 2333 | CatBoost | 14 routinely available predictors selected by recursive feature elimination | Internal test cohort and external validation cohort | AUC 0.819 internal and 0.807 external | Retrospective design; external validation limited to Chinese medical-centre data; prospective utility not established |

Abbreviations: AI, artificial intelligence; ANN, artificial neural network; ALT, alanine aminotransferase; ApoB, apolipoprotein B; AUC, area under the receiver operating characteristic curve; BMI, body mass index; BPRP, Beijing Prediabetes Reversion Program; CGM, continuous glucose monitoring; CRP, C-reactive protein; DPP, Diabetes Prevention Program; DT, decision tree; EHR, electronic health record; FPG, fasting plasma glucose; HDL-C, high-density lipoprotein cholesterol; HbA1c, glycated haemoglobin; iIGT, isolated impaired glucose tolerance; LASSO, least absolute shrinkage and selection operator; LR, logistic regression; MESA, Multi-Ethnic Study of Atherosclerosis; ML, machine learning; NB, naive Bayes; NPV, negative predictive value; OGTT, oral glucose tolerance test; PPV, positive predictive value; RF, random forest; SHAP, SHapley Additive exPlanations; SVM, support vector machine; T2DM, type 2 diabetes mellitus; TC, total cholesterol; TG, triglycerides; WBC, white blood cell count; XGBoost, extreme gradient boosting.

**Table 2. Summary of digital, algorithm-driven, and AI-assisted lifestyle intervention studies in prediabetes**

| **Study** | **Design and population** | **Intervention type** | **AI or algorithmic component** | **Follow-up** | **Main outcomes** | **Key limitations** |
| --- | --- | --- | --- | --- | --- | --- |
| Block et al. / Alive-PD | Randomised controlled trial in adults with laboratory-confirmed prediabetes in a large U.S. health system | Fully automated, algorithm-driven behavioural intervention delivered by email, web portal, and mobile phone | Rule-based computer algorithms for personalised weekly goals, feedback, and problem-solving tips | 6 months | Greater reductions in HbA1c, FPG, body weight, BMI, waist circumference, TG/HDL-C ratio, and predicted diabetes risk | Primarily rule-based rather than ML-based adaptation; comparator was usual care; long-term durability uncertain |
| Kitazawa et al. | Randomised controlled trial in adults at high risk of T2DM | Smartphone app plus intermittently scanned CGM | Sensor-enabled and rule-based feedback using CGM traces, diet, and physical activity information | 12 weeks | Improved CGM-derived glycaemic metrics, modest weight loss, and better self-reported lifestyle behaviours | Feedback algorithms were mainly rule-based; short follow-up |
| Everett et al. / Sweetch | Pilot clinical trial in adults with prediabetes | Fully automated mobile health coaching platform | Proprietary ML algorithm tailoring frequency, timing, and content of push notifications according to baseline risk, preferences, engagement, and activity patterns | 3 months | Increased physical activity, clinically meaningful weight loss, modest HbA1c reduction, and engagement-related outcome improvement | Pilot design; no randomised control group; short follow-up; limited causal inference |
| Cakmak et al. / TH-001 | 90-day randomised controlled trial in adults with prediabetes | Digital therapeutic delivered through mobile application | Structured lifestyle content, personalised prompts, and self-monitoring tools; degree of adaptive ML personalisation requires clarification | 90 days | Greater reductions in HbA1c and body weight; higher engagement associated with greater glycaemic improvement | Mechanism appears mainly digital behavioural support unless adaptive ML is demonstrated; short duration; long-term outcomes unknown |
| Mathioudakis et al. | Randomised clinical trial in adults with prediabetes and overweight or obesity | AI-powered DPP-based lifestyle intervention delivered through mobile app and connected digital scale | Adaptive algorithms derived from Sweetch platform to personalise goals, messages, and feedback without ongoing human coaching | 12 months | Non-inferior to human coaching for composite outcome; similar endpoint achievement, 31.7% vs 31.9%; engagement at least as high as human coaching | Modest sample size; single health system; one-year follow-up; longer-term durability and broader generalisability uncertain |

Abbreviations: AI, artificial intelligence; BMI, body mass index; CGM, continuous glucose monitoring; DPP, Diabetes Prevention Program; FPG, fasting plasma glucose; HbA1c, glycated haemoglobin; HDL-C, high-density lipoprotein cholesterol; ML, machine learning; T2DM, type 2 diabetes mellitus; TG, triglycerides.
